# Dysregulated Forkhead Box (FOX) Genes Association with Survival Prognosis, Anti-tumor Immunity, and Key Targeting Drugs in Colon Adenocarcinoma

**DOI:** 10.34172/aim.2023.77

**Published:** 2023-09-01

**Authors:** Qian Xie, Jie Wang, Xingchen Peng

**Affiliations:** ^1^International Medical Center/Ward of General Practice, West China Hospital, Sichuan University, Chengdu, 610000, China; ^2^Department of Pharmacy, First Affiliated Hospital of Xinjiang Medical University, Urumqi, 830011, China; ^3^Department of Biotherapy, Cancer Center, West China Hospital, Sichuan University, Chengdu, 610000, China

**Keywords:** Colon adenocarcinoma, Cancer-associated pathways, FOX genes, Immune responses, Molecular docking

## Abstract

**Background:**

Several studies have revealed that the aberrant expressions of forkhead box (FOX) genes are associated with carcinogenesis. However, the crucial biological functions of the FOX gene in colon adenocarcinoma (COAD) remain unknown.

**Methods:**

The TCGA-COAD dataset (n=328) was utilized for determining the deregulated FOX genes and their association with functional enrichment, protein-protein interaction (PPI), survival prognosis, anti-tumor immunity, cancer-associated pathways, and biological processes in COAD. In addition, we used GSE166427 (GPL13667) as a validation cohort (n=196). Molecular docking studies were applied to perform the drug interactions.

**Results:**

The FOX genes are deregulated in the COAD (Log_2_ FC>0.50, *P*<0.05), and the PPI network of FOX members is substantially related to the enrichment of cancerous signaling, immune responses, and cellular development (FDR<0.05). A worse prognosis for overall survival in COAD individuals is connected with the subgroup of FOX transcripts (*P*≤0.05). *FOXD4*, *FOXH1*, and *FOXS1* were identified as predictive variables in the univariate and multivariate Cox regression models (*P*≤0.05). *FOXH1* and *FOXS1* are substantially linked to the deregulated immunity in COAD (R>0.20, *P*<0.01). Furthermore, *FOXS1* expression regulates cancer-associated pathways and biological processes (*P*<0.05). Moreover, *FOXD4*, *FOXH1*, and *FOXS1* are genetically altered and showed diagnostic efficacy in COAD. We revealed that *FOXD4*, *FOXH1*, and *FOXS1* are consistently deregulated in GSE166427 (*P*<0.05). Finally, molecular docking revealed that *FOXH1* interacted with various drugs, including belinostat, entinostat, and panobinostat.

**Conclusion:**

The FOX genes have a strong correlation with the poor prognosis for survival, tumor immunity, cancer-associated pathways, and biochemical processes that cause the pathogenesis of COAD.

## Introduction

 In the human genome, there are more than 50 forkhead box (FOX) genes or proteins, divided into 19 subclasses (*FOX*A to *FOXS*) based upon sequence homology inside and outside the forkhead domain.^1,2^ Various FOX genes regulate crucial physiological processes, including embryogenesis, cellular homeostasis, and the immune system.^3,4^ The functioning of the lung, kidney, immune function, and nerves is also regulated by the FOX genes.^3,4^ The FOX genes promote and collaborate with certain other active gene transcription and epigenetic controllers, acting as transcriptional activators, transcriptional repressors, and pioneer factors.^2^ It has been reported that the FOX genes and their translated protein are involved in the development, metabolism, cancer, and aging.^2,4^ Cancer genesis, growth, maintenance, advancement, and medication resistance are all closely correlated with dysfunctional FOX genes.^2^ Also, FOX genes are associated with substantial cancerous biological processes, including senescence, apoptosis, DNA damage repair, and survival of cancer patients.^2,5^ Various FOX genes are related to tumor-promoting inflammation, immune destruction, genome instability and mutation, angiogenesis, proliferative signaling, tumor invasion, metastasis, and resisting cell death.^3^

 Malignancies in several tissues, such as the lung, breast, and thyroid, have been linked to the amplification of FOX genes.^6^ FOX genes act downstream of several oncogenic signaling pathways, including PI3K–AKT, ERK, Wnt, β-catenin, EGFR, and NF-κB-IKKβ, associated with CRC cancerogenesis.^7^ The clinical results of cancer patients are strongly correlated with deregulated FOX genes. For example, Shan et al discovered that nasopharyngeal cancer patients have a poor prognosis when their *FOXJ2* (OMIM number: 619162) activity is increased.^8^ According to Song et al, the crucial predictive factor for overall survival (OS) in those with various malignancies is *FOXM1* (OMIM number: 602341).^9^ The *FOXD1* (OMIM number: 601091) protein was significantly elevated in the primary HNSCC cohort. Significant associations existed between its abundance, cervical node metastases, and poor overall and disease-free survival (DFS).^10^ In colorectal cancer, the expression of *FOXM1* correlates with poor prognosis.^11^ In addition to the clinical outcomes, FOX genes are critically associated with immunoregulatory functions. The FOX genes are associated with immunoregulation, including regulating CD4 + T cell tolerance, thymic development, macrophage differentiation, natural killer cell effector function, and T cell activation.^4^ The *FOXO*s regulate the immune system by modulating tumor and stromal cells.^12^ Martin et al showed that the *FOXP3* (OMIM number: 300292) is an immunosuppressive marker in human malignancies.^13^ These studies provided crucial information about the oncogenic roles of FOX genes associated with cancer onset, development, metastasis, tumor immunity, and signaling pathways in human malignancies.

 Globally, colon cancer is the second most common cause of mortality (9.4%), and the third most commonly diagnosed disease (10.0%).^14^ Here, we present a comprehensive bioinformatics study to evaluate the changes in FOX gene expression levels. Also, we identified the functional enrichment of deregulated FOX genes in colon adenocarcinoma (COAD). Additionally, we investigated the involvement of FOX genes in protein-protein interactions (PPIs) and their association with the enrichment of pathways. We further examined the connection between altered FOX genes and the survival rates of COAD individuals. Univariate, multivariable, and nomogram analysis revealed COAD’s independent prognostic FOX genes. Then, we identified that the prognostic FOX genes were related to the immune content, stromal content, purity of tumor, immune signatures, immune ratio, cancer-associated signaling pathways, and cancer hallmark biological processes. Finally, we observed that the prognostic FOX genes were genetically altered and effective in diagnosing COAD individuals.

## Materials and Methods

###  Datasets

 We transformed the data into a log2-base conversion after downloading the TCGA COAD dataset from https://portal.gdc.cancer.gov. We utilized relevant information from the TCGA COAD cohort to compare the survivorship of two patient groups in the database (https://portal.gdc.cancer.gov) (n = 328, 287 tumor samples, and 41 normal samples). In addition, we investigated the transcript levels of FOX genes in multiple types of cancer using the Oncomine repository (https://www.oncomine.org/resource/login.html). To determine the genetic changes in three prognostic FOX genes, we utilized COAD (TCGA, Firehose Legacy) individuals with mutation and CNA information (n = 220) in cBioPortal (http://www.cbioportal.org/). Finally, we used GSE166427 (GPL13667, 98 tumor samples, and 98 normal samples) as a validation cohort (n = 196).^15^

###  Investigations of FOX Gene Variations in COAD Compared to the Control

 The Limma linear model, which has a range of test conditions and predictors, is specifically made for assessing complicated studies. When comparing COAD samples (n = 287) to control samples (n = 41), we used the R package “limma” to find the relevant DEGs.^16^ Utilizing the TCGA COAD cohort, we identified the significant FOX genes in the COAD individuals relative to the control sample.

###  Gene-Set Enrichment Analysis 

 A computational technique called gene-set enrichment analysis (GSEA) is capable of helping assess if a predefined gene collection exhibits statistically meaningful, concordant changes between two biological contexts. Using the GSEA tool, we investigated the GSEA of the candidate genes.^17^ We entered the gene set into the GSEA tool to find the significant GO and pathways. The Reactome pathways^18^ significantly associated with the FOX genes were identified.

###  Protein-Protein Interaction Network Construction

 Using the NetworkAnalyst application, we developed a PPI network of the DEGs.^19^ In the NetworkAnalyst software, we used the STRING tool^20^ by selecting interactome with a medium interactome score (400) and confidence score cutoff of 900, and required experimental evidence was also determined. The node explorer module of NetworkAnalyst was used to find the ranking genes (those with a high degree of connectivity to specific other nodes) in the PPI network.^19^ To find KEGG pathways connected to generated PPI, we used the function explorer module of the NetworkAnalyst tool. Using the software Cytoscape 3.8.2, we illustrated the PPI networks.^21^

###  Survival Analysis of FOX Genes in COAD

 In the two groups of individuals with colon cancer, we evaluated the OS and the DFS. According to our assessment of the clinical information from the TCGA COAD cohort, the follow-up period was 147.9 months. The survival disparities between the high-expression group (HEG) and low-expression group (LEG) of FOX genes in COAD individuals were identified using the Kaplan-Meier method (HEG > median > LEG). Utilizing the R package “survival”, the survival implications of significant FOX genes in the TCGA COAD database were investigated.^22^ We used the “coxph” function in the R package “survival” for the univariate and multivariable Cox regression assessments of variables. The nomogram was generated using the R package “rms”.^22^

###  ESTIMATE Algorithm for Quantifying Immune Score, Stromal Score, and Tumor Purity

 The R-based ESTIMATE program predicts the tumor purity, immune score, and stromal score. This technique utilizes the gene expression patterns of 141 stromal genes and 141 immune genes.^23^ Applying relevant gene expression matrix information, the quantity of stromal cells and immune cells infiltrating into malignant cells was determined.^23^ Next, we contrasted the immune, stromal, and tumor purity values between the HEG and LEG of crucial FOX genes (Wilcoxon sum rank test, *P* < 0.05).

###  Single Sample Gene Set Enrichment Analysis 

 Instead of using a sample population, the single sample gene set enrichment analysis (ssGSEA) approach operates on a single-sample basis. For every pairing of samples and genes set in the cancer tissues, we used the extended modules of GSEA and ssGSEA to determine the enrichment levels of immune cells, cancer-associated pathways, and hallmark biological processes.^24^ To calculate the ssGSEA scores of particular immune signatures, pathways, and biological processes, we assembled the marker gene sets.^25-28^ We identified the ssGSEA score of various immune signatures (such as CAFs, HLA genes, immune cell infiltrate genes, immune checkpoint genes, pDCs, etc), cancerous KEGG pathways (such as cell cycle, JAK-STAT, MAPK, mTOR, WNT, etc),^29^ and three key hallmark biological processes (epithelial-mesenchymal transition [EMT], angiogenesis, and hypoxia). The relationships between the enrichment levels (ssGSEA scores) of immunological signatures, pathway activity, and biological processes and the levels of FOX gene expression were investigated using Spearman’s correlation test. The marker gene sets with immunological signatures, pathway activity, and biological processes are listed in Table S1 (see Supplementary file 1).

###  Analysis of Genetic Changes in Prognostic FOX Genes

 Utilizing cBioPortal, an open-access platform for assessing genetic modifications in multimodal cancer studies, we were able to determine the genetic changes in independent prognostic FOX genes. In our work, we identified the genetic of prognostic FOX genes using the COAD (TCGA, Firehose Legacy) individuals with mutation and CNA data (n = 220) in cBioPortal (http://www.cbioportal.org/).

###  Diagnostic Effectiveness of Prognostic FOX Genes in COAD

 To properly evaluate ROC curves and partial areas under the curve, the “pROC” R package provides an assortment of statistical models. The area under the ROC curve (AUC) was determined using the “pROC” R package to assess the capacity to discriminate between COAD and healthy samples. The graph was shown to determine the diagnostic values of prognostic FOX genes.^30^ The AUC values represented the diagnostic value and differences between tumor and healthy samples for each gene, and the AUC > 0.5 for a single gene was defined as the diagnostic efficacy.^31^

###  Validation of Key Gene Expression in an Independent Dataset

 As a validation cohort, we used GSE166427 (GPL13667) to confirm the expression levels of critical genes (n = 196, 98 tumor samples, and 98 normal samples).^15^ This database aimed to evaluate the expression profiling of COAD and normal adjacent colon cells. The platform GPL13667 is based on Affymetrix Human Genome U219 Array. We downloaded the series matrix data and utilized the t-test to identify the DEGs between colon tumors and adjacent normal colon cells. We selected the genes with the highest value of fold change for multiple probes of a single gene.

###  Exploration of the Drug Compound’s Interaction with Key Genes and their Molecular Docking 

 We employed the NetworkAnalyst^19^ software for extracting the chemical-protein interaction. We inputted the gene symbol of key genes into the NetworkAnalyst^19^ software and identified the networks or sub-networks between the genes and chemical compounds. We utilized the Cytoscape tool to show the retrieved drug-gene interaction. Then, we used these interacting compounds for molecular docking analysis. We downloaded the protein product of *FOXH1 *(OMIM number: 603621) (protein database ID: 5XOC) and all other chemical compounds interacting with this gene. We prepared the protein using Discovery studio (https://3ds.com/products-services/biovia/products). First, we eliminated ligands and water molecules from the receptor proteins. Next, we prepared the ligand using PyRx (https://pyrx.sourceforge.io/). Finally, using PyRx, we conducted a molecular docking investigation (https://pyrx.sourceforge.io/).

###  Statistical Analysis

 Log2FC > 0.50 (absolute value) and *P* < 0.05 were the cutoffs we used to identify the DEGs. For choosing the significantly enriched GOs and pathways, the FDR < 0.05 was considered. When comparing the two patient groups’ survival rates using Cox regression, a *P* ≤ 0.05 was considered significant. The Wilcoxon sum rank test was performed to contrast the two patient groups (*P* < 0.05). To determine the significance levels between the two factors, we used either Pearson’s or Spearman’s correlation test. The levels of FOX genes and the ssGSEA scores of immunological signatures, pathways, and biological processes were analyzed using Spearman’s correlation test because the data were not normally distributed (*P* < 0.05). We used Pearson’s correlation test to examine the relationships between the expression levels of FOX genes and the expression levels of other immune-marker genes and FOX genes since the data were normally distributed (*P* < 0.05). During the validation of key genes in the GSE166427, the *P* < 0.05 cutoff was established as the significant value.We used the R package “ggplot2” to present the information from our investigation.

## Results

###  Identifying the Differentially Expressed FOX Genes in the COAD

**Figure 1:**
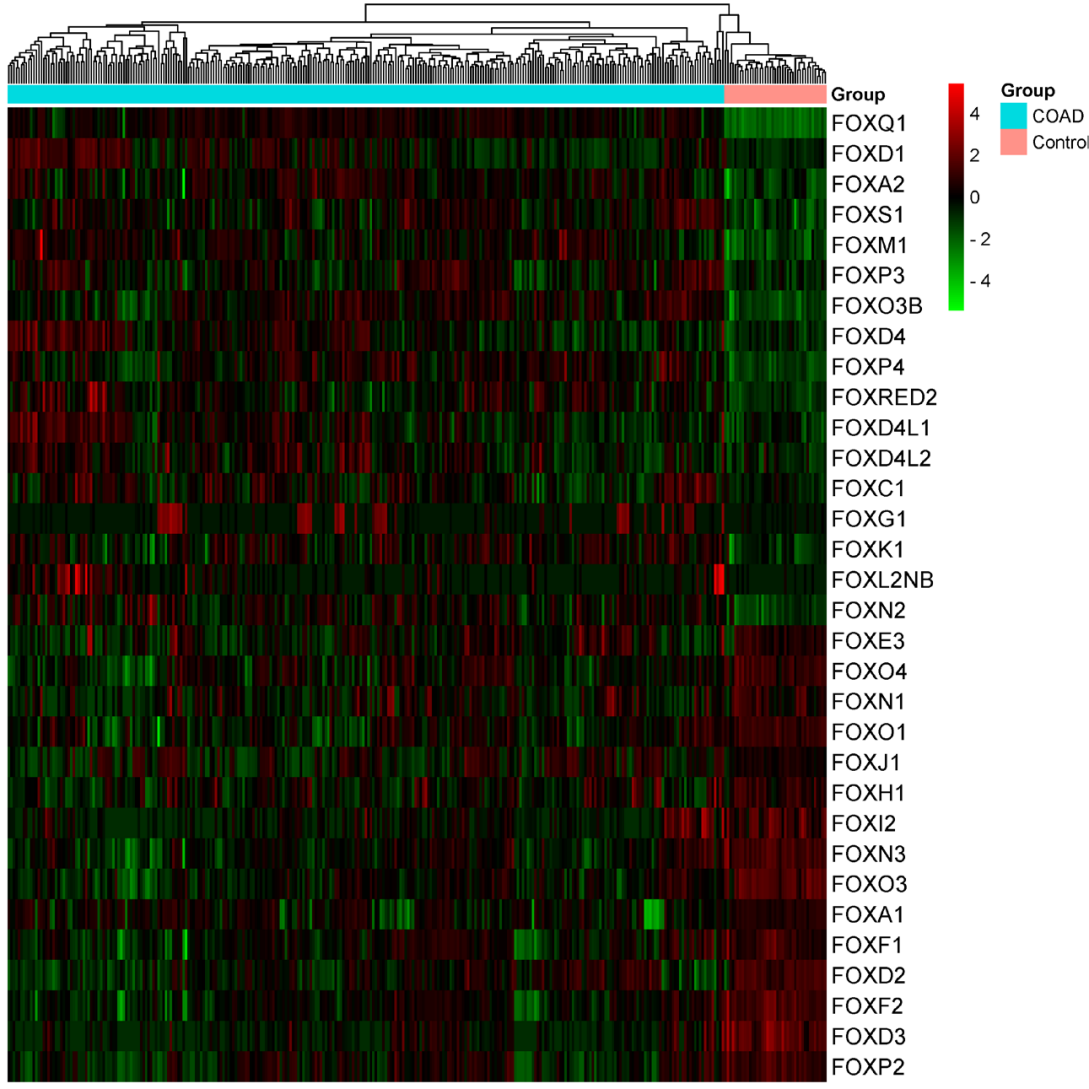
Heatmap of the 32 Differentially Expressed FOX Genes, Including 17 Upregulated and 15 Downregulated in COAD Individuals

 We analyzed the COAD’s significantly differentially expressed FOX genes relative to the normal samples (Log_2_FC > 0.50, Adjusted *P*< 0.0001). We found that 17 FOX genes have increased expression levels in COAD (Table 1). In contrast, 15 FOX genes exhibited a downregulation trend in the COAD (Table 1). Figure 1 shows the heatmap of the genes’ transcriptional value with differential expression. After passing the threshold, the expression of additional FOX genes did not change in the TCGA COAD samples.

**Table 1:** Differential Expression of FOX Genes in TCGA COAD

**Entrez ID**	**Log**_2_** FC**	**Regulatory Status**	**Mean Expression level**	***P *** **Value**	**Adjusted ** ***P *** **Value**	**Symbols of genes**	**Name of Genes (OMIM#)**
94234	5.86	Up regulated	8.8838	1.62E-76	6.95E-75	*FOXQ1*	forkhead box Q1 (612788)
2297	1.91		3.9676	5.79E-06	9.96E-06	*FOXD1*	forkhead box D1 (601091)
3170	1.56		9.3515	5.76E-18	2.75E-17	*FOXA2*	forkhead box A2 (600288)
2307	1.51		5.0226	2.09E-16	6.92E-16	*FOXS1*	forkhead box S1 (602939)
2305	1.39		10.434	4.36E-27	4.24E-26	*FOXM1*	forkhead box M1 (602341)
50943	1.22		5.1661	3.03E-07	5.70E-07	*FOXP3*	forkhead box P3 (300292)
2309	1.14		10.417	2.57E-24	1.58E-23	*FOXO3*	forkhead box O3 (602681)
2298	1.11		2.9289	3.02E-08	6.18E-08	*FOXD4*	forkhead box D4 (601092)
116113	0.90		11.378	5.18E-20	2.78E-19	*FOXP4*	forkhead box P4 (608924)
80020	0.90		8.7531	1.83E-12	4.14E-12	*FOXRED2*	FAD-dependent oxidoreductase domain containing 2 (613777)
200350	0.84		2.1282	4.59E-07	8.23E-07	*FOXD4L1*	forkhead box D4 like 1 (611084)
100036519	0.69		2.2128	4.44E-06	4.94E-06	*FOXD4L2 *	forkhead box D4 like2 (611086)
2296	0.66		6.1097	0.006004	0.008328	*FOXC1*	forkhead box C1 (601090)
2290	0.61		0.87147	0.03668	0.045064	*FOXG1*	forkhead box G1 (164874)
221937	0.58		10.438	6.08E-09	1.31E-08	*FOXK1*	forkhead box K1 (616302)
401089	0.56		0.62423	0.002925	0.004192	*FOXL2NB*	FOXL2 neighbor*
3344	0.52		9.0173	1.18E-14	3.62E-14	*FOXN2*	forkhead box N2 (143089)
2301	-0.76	Down regulated	1.8929	0.000202	0.000299	*FOXE3*	forkhead box E3 (601094)
4303	-0.85		9.1094	1.99E-14	5.34E-14	*FOXO4*	forkhead box O4 (300033)
8456	-0.95		1.6649	1.16E-05	1.85E-05	*FOXN1*	forkhead box N1 (600838)
2308	-0.99		9.0698	2.18E-13	5.51E-13	*FOXO1*	forkhead box O1 (136533)
2302	-1.07		4.7494	0.027403	0.035952	*FOXJ1*	forkhead box J1 (602291)
8928	-1.15		3.8509	3.05E-07	5.70E-07	*FOXH1*	forkhead box H1 (603621)
399823	-1.17		0.8496	5.08E-17	1.99E-16	*FOXI2*	forkhead box I2 (617202)
1112	-1.20		10.146	2.03E-24	1.45E-23	*FOXN3*	forkhead box N3 (602628)
2310	-1.31		9.4959	2.04E-32	2.93E-31	*FOXO3B*	forkhead box O3B*
3169	-1.31		8.6048	7.02E-06	1.16E-05	*FOXA1*	forkhead box A1(602294)
2294	-1.49		8.0854	2.53E-13	6.05E-13	*FOXF1*	forkhead box F1 (614975)
2306	-1.58		7.6406	7.41E-17	2.65E-16	*FOXD2*	forkhead box D2 (602211)
2295	-2.48		6.5547	4.92E-27	4.24E-26	*FOXF2*	forkhead box F2 (603250)
27022	-2.99		1.2901	1.79E-45	3.86E-44	*FOXD3*	forkhead box D3 (611539)
93986	-3.11		4.3298	9.00E-18	3.87E-17	*FOXP2*	forkhead box P2 (605317)

The character “OMIM#” is the OMIM number obtained from the website https://www.omim.org/. * indicates that the OMIM number was not found on the website (https://www.omim.org/).

###  Association of FOX Genes with Pathway Deregulation and Functional Enrichment

**Figure 2:**
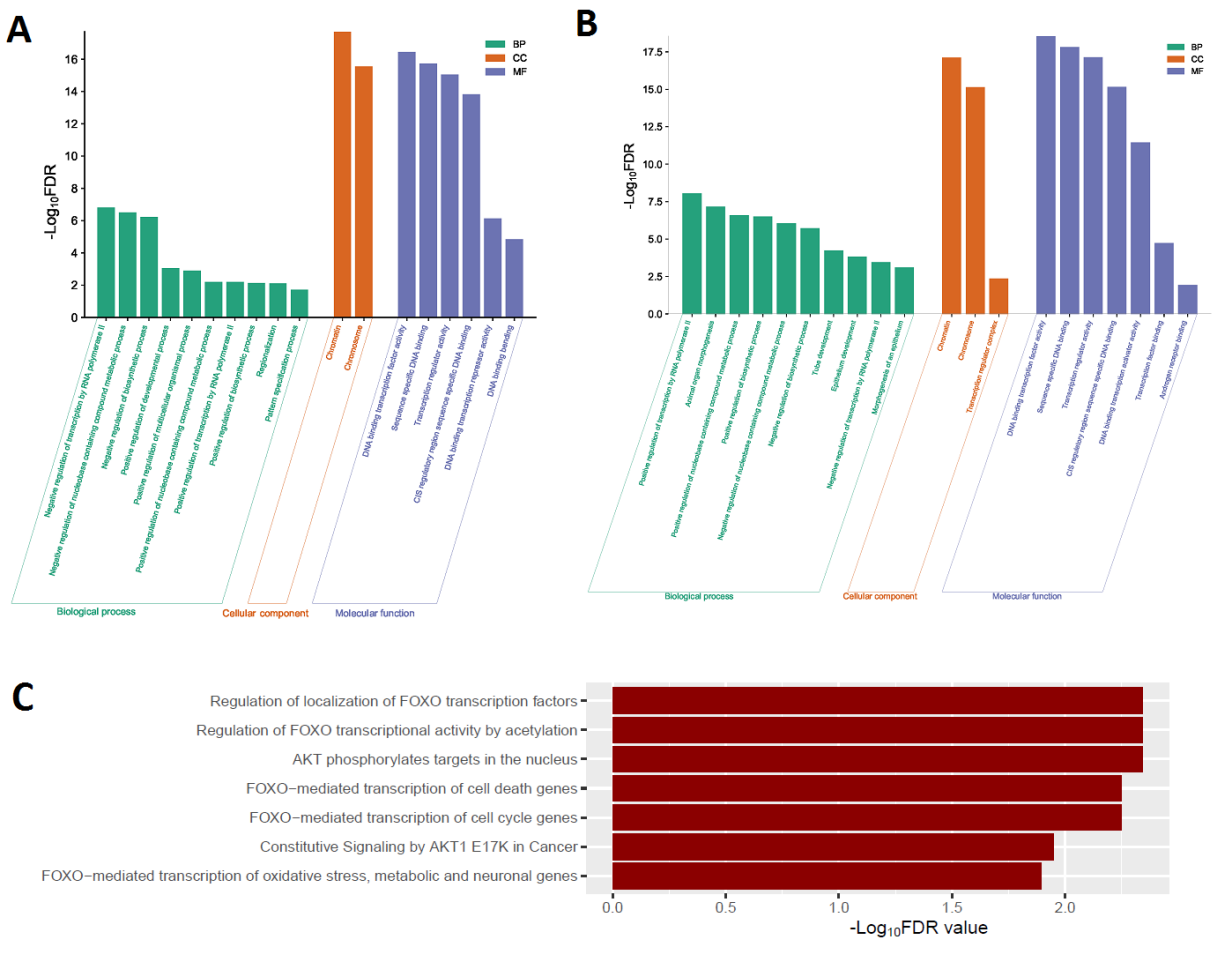
Significantly Enriched GO and Reactome Pathways Associated with FOX Genes.** A.** The enriched GOs are associated with upregulated FOX genes in COAD.** B. **The enriched GOs are associated with downregulated FOX genes in COAD.** C.** Downregulated FOX genes in COAD regulate the significantly enriched Reactome pathways

 We utilized the GSEA tool to identify the enriched gene ontology (GO) and deregulated pathways significantly linked to the dysfunctional FOX genes (Figure 2). We identified biological processes (such as regionalization, negative regulation of DNA binding transcription factor activity, and positive regulation of developmental process) that strongly correlate to increased FOX genes (Figure 2A). The cellular components, including chromatin and chromosome, are enriched considerably with upregulated FOX genes (Figure 2A). Moreover, we found the six molecular functions (such as DNA binding bending, DNA binding transcription repressor activity, and transcription regulator activity) that strongly correlate to increased FOX genes (Figure 2A). In addition, the downregulated FOX genes enrich GO terms and signaling pathways. We identified 63 biological processes (such as positive regulation of the biosynthetic process, animal organ morphogenesis, and tube development) that are significantly associated with downregulated FOX genes (Figure 2B and Table S2). The chromatin, chromosome, and transcription regulator complex are significantly enriched cellular components related to the decreased FOX genes (Figure 2B). Moreover, we found seven enriched considerably molecular functions related to the decreased FOX genes (Figure 2B).

 Furthermore, we revealed the deregulated Reactome pathways associated with differentially expressed FOX genes. We found that the Reactome *FOXO* mediated transcription of cell cycle genes pathway is significantly enriched with upregulated FOX genes. In addition, we found seven pathways (such as AKT phosphorylates targets in the nucleus, regulation of *FOXO* transcriptional activity by acetylation, and regulation of localization of *FOXO* transcription factors) that are significantly associated with downregulated FOX genes in COAD (Figure 2C). It suggests that COAD transcriptional activity and other carcinogenic signaling mechanisms are correlated to uncontrolled FOX genes.

###  FOX Genes Associated with PPI Network and Correlated with Each Other

**Figure 3:**
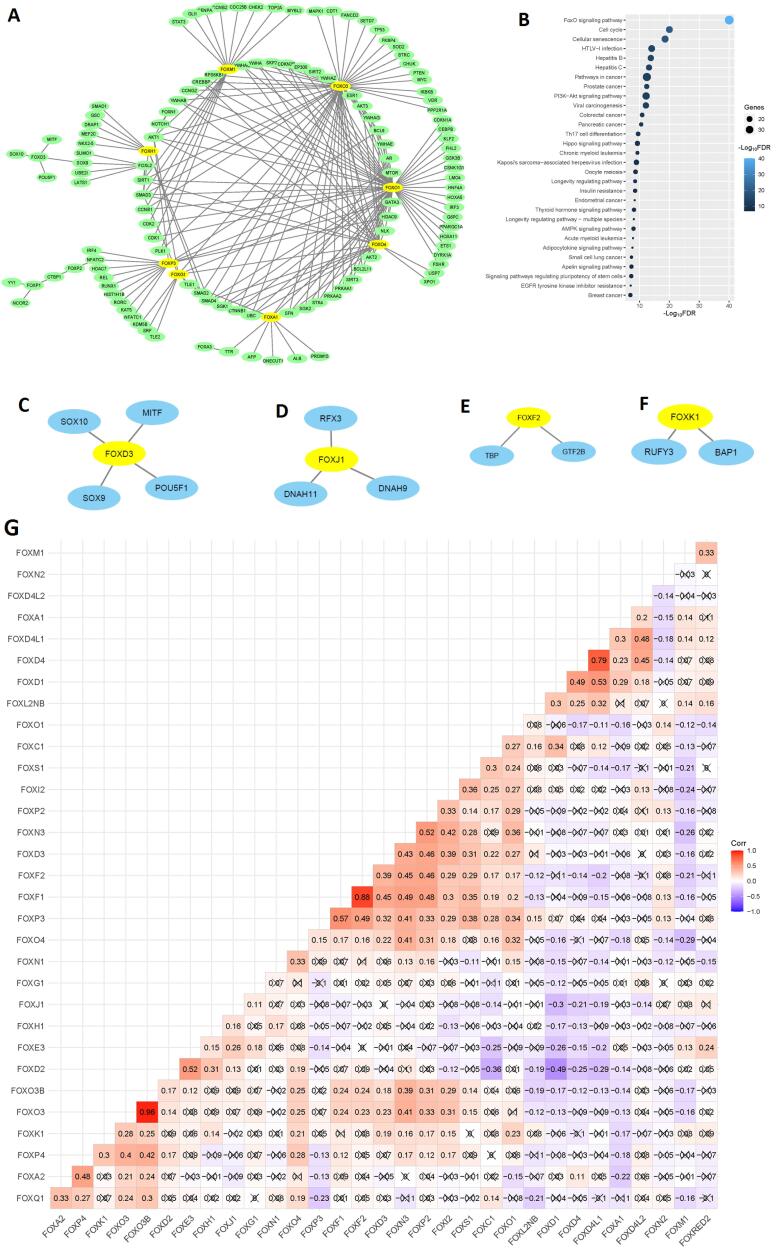
Involvement of FOX Genes in the PPI Network and the Relationship of FOX Genes with Other Family Members.** A.** Involvement of FOX genes in the PPI (sub-network 1) network. **B.** The top 30 KEGG pathways are considerably enriched and connected to sub-network 1. **C-F.** The other four sub-networks of FOX genes in the PPI. **G.** Relationship between FOX genes in the TCGA COAD data (Pearson correlation test). × represents the non-significant value (*P* > 0.05)

 We inputted deregulated FOX genes (Table 1) into the NetworkAnalyst software to construct the PPI network. In the NetworkAnalyst software, we used the STRING tool^20^ with a medium interactome score (400), and confidence score cutoff of 900, and the required experimental evidence was also selected. In the PPI, we found that the FOX genes are associated with five sub-networks. In sub-network 1, the eight FOX genes, including *FOXO1, FOXO3, FOXO4, FOXM1, FOXP3, FOXG1, FOXA1, *and* FOXH1* are involved in the PPI network (Figure 3A). All nodes in sub-network 1 are listed in Table S3. Interestingly, the function module of the NetworkAnalyst software identifies that sub-network 1 is substantially related to the enriched 95 KEGG pathways (Table S4). The top 30 pathways are illustrated in Figure 3B. These significant (FDR < 0.05) pathways mainly involved with cancer (such as pathways in cancer, endometrial cancer, colorectal cancer, small cell lung cancer, and central carbon metabolism in cancer), immunity (such as Th17 cell differentiation, B cell receptor signaling pathway, and T cell receptor signaling pathway), and cellular signaling and development (such as TGF-beta signaling pathway, cell cycle, and cellular senescence). It suggests that sub-network 1 is related to the regulation of tumor immunity and tumor biology in COAD. In sub-network 2, the *FOXD3* interacts with four genes (Figure 3C). In other sub-networks, *FOXJ1, FOXF2, *and* FOXK1* are involved with the PPI network (Figure 3D-F). After determining the PPI of the FOX genes, we hypothesized that the FOX genes are interconnected (Pearson’s correlation test, *P* > 0.05). Interestingly, we found that some FOX genes correlate with each other (Figure 3G). For example, there is a substantial positive correlation between *FOXP3* and *FOXF1, FOXF2, FOXD3, FOXN3, FOXP2, FOXI2, FOXS1, FOXC1, *and* FOXO1 *levels (Figure 3G). Similarly, the expression level of *FOXS1* has a significant positive correlation with the expression of *FOXO1, FOXI2, FOXO4, FOXP3, FOXF1, FOXF2, *and* FOXD*3 (Figure 3G). In contrast, *FOXD2* negatively correlates with the expression level of *FOXC1, FOXO1, FOXD1, FOXD4, *and *FOXD4L1 *(Figure 3G). It indicates that the FOX genes may be regulating the expression of each other and contribute to the carcinogenesis of COAD.

###  Dysfunctional FOX Genes Related to Poor Survival Prognosis in the COAD

**Figure 4:**
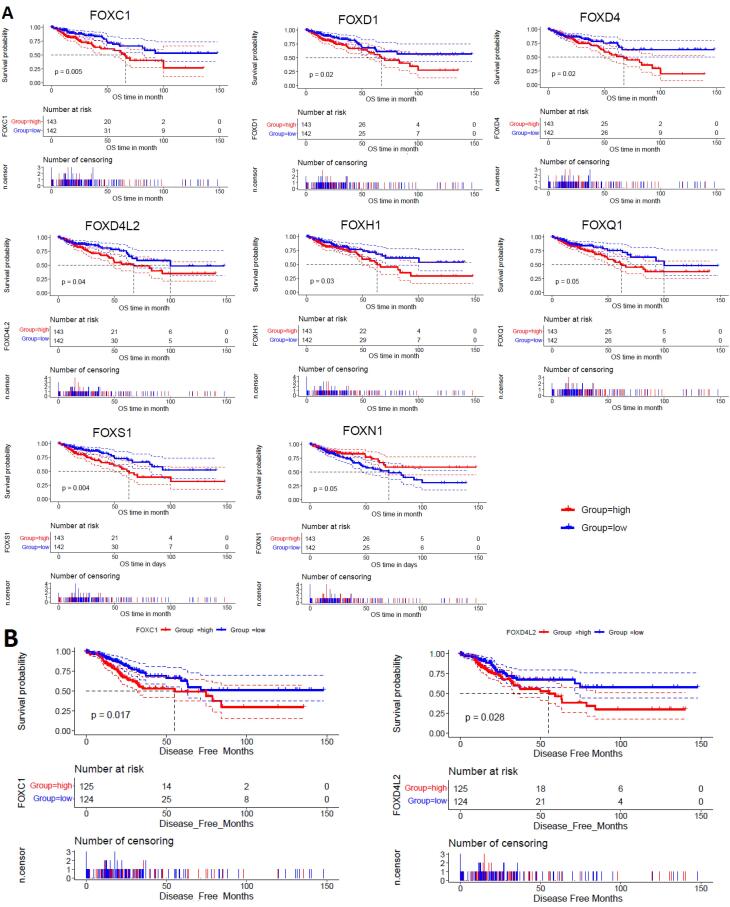
Exploration of Prognostic FOX Genes in the COAD.** A. **The HEG patients of *FOXC1, FOXD1, FOXD4, FOXD4L2, FOXH1, FOXQ1, *and *FOXS1* showed a substantially poor OS rate in COAD. The LEG patients of *FOXN1 *showed a substantially poor OS rate in COAD. **B. **The HEG patients of *FOXC1* and *FOXD4L2* showed a substantially shorter DFS rate in COAD

 We investigated the survival prognosis of dysfunctional FOX genes (shown in Table 1) using the clinical information of the TCGA COAD cohort. We discovered that patients with COAD had a worse OS prognosis when the expression level of *FOXC1, FOXD1, FOXD4, FOXD4L2, FOXH1, FOXQ1, *and* FOXS1* is higher than the median (HEG) (Figure 4A). In contrast, the higher expression group (HEG > median) of *FOXN1* has a favorable survival prognosis in COAD patients (Figure 4A). In addition, the higher expression group (HEG > median) of *FOXC1 *and* FOXD4L2* are poorly linked with DFS time in COAD patients (Figure 4B).

**Figure 5:**
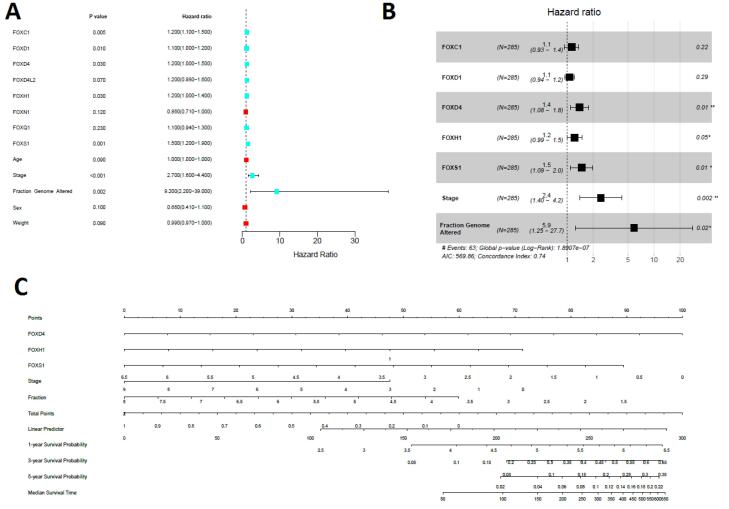
Univariate and Multivariable Analysis of OS for Identifying the Prognostic Factors Associated with Nomogram Prediction.** A.** The univariate Cox model explored the risk factors in COAD, including *FOXC1, FOXD1, FOXD4, FOXH1, *and* FOXS1*. **B. **The multivariable Cox found that the stage, fraction genome altered, *FOXD4, FOXH1, *and* FOXS1* are risk factors in COAD. **C. **Identifying the risk factors in the constructed nomogram. The nomogram predicted COAD’s 1, 2, and 3 years OS. Fraction = fraction genome alterations

 Moreover, we applied univariate Cox regression analyses of the expression of seven prognostic genes (*FOXC1, FOXD1, FOXD4, FOXD4L2, FOXH1, FOXQ1, *and* FOXS1*), age, sex, weight, stage, and genome fraction altered. The univariate Cox regression analyses identified five genes (*FOXC1, FOXD1, FOXD4, FOXH1, *and *FOXS1*), stage, and fraction genome alteredas significant prognostic factors (Figure 5A). Furthermore, we investigated the multivariable Cox model with the levels of *FOXC1, FOXD1, FOXD4, FOXH1, *and* FOXS1* genes, with COAD stage (stage I and stage II versus stage III and stage IV), and fraction genome altered as the predictor variables. Remarkably, we discovered that stage, fraction genome alterations, and the levels of three genes (*FOXD4, FOXH1, *and* FOXS1*) were the independent prognostic variables (Figure 5B). Finally, the nomogram was constructed to determine the possibility of these three prognostic FOX genes and stages of the tumor with fraction genome alterations influencing the prognostic outcome (Figure 5C).

###  The Dysfunctional Prognostic FOX Genes Regulating the Tumor Immunity in the COAD

**Figure 6:**
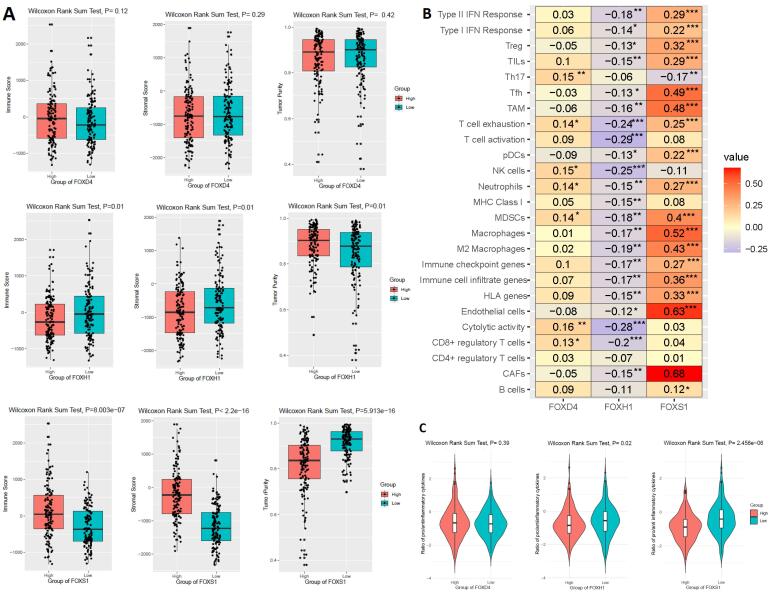
Prognostic FOX Genes Correlated with the Regulation of Immunity in the COAD.** A. **Immune scores, stromal scores, and tumor purity are deregulated with the expression of prognostic FOX genes**. B. **The correlation of various immune cells with the prognostic FOX genes in the COAD. **C. **The ratios of pro-/anti-inflammatory cytokines were lower in the HEG patients compared to the LEG patients (Wilcoxon sum rank test, P < 0.005). We used the *IFNG*, *IL-1A*, *IL-1B*, and *IL-2 *marker genes as the pro-inflammatory cytokines and the *IL-4*, *IL-10*, *IL-11*, and *TGFB1* marker genes as the anti-inflammatory cytokines^27^. * is *P*< 0.05, ** is *P*< 0.01, and *** is *P* < 0.001

 In human CRC, the level of tumor-infiltrating lymphocytes (TILs) is a remarkable indicator of eventual survival.^32^ We investigated how the prognostic FOX markers *FOXD4, FOXH1, *and *FOXS1* correlated with the immune scores, stromal scores, tumor purity, and TIL quantities in the TCGA COAD. First, we found that the stromal scores, immune scores, and tumor purity are significantly deregulated (Wilcoxon sum rank test, *P* < 0.05) between the HEG and LEG of *FOXH1 *and* FOXS1* (Figure 6A). However, the expression of *FOXD4 *is not significantly associated with these immune factors (Wilcoxon sum rank test, *P* < 0.05) (Figure 6A). Second, we investigated the correlation (Spearman correlation test, R > 0.20, *P* < 0.01) of these three independent prognostic factors (*FOXD4, FOXH1, *and* FOXS1*) with several immune signatures (Figure 6B). Our analysis revealed that *FOXS1* level showed a positive relationship with different immune signatures, such as CAFs, endothelial cells, HLA genes, immune cell infiltrate genes, immune checkpoint genes, macrophages, M2 macrophages, MDSCs, neutrophils, pDCs, T cell exhaustion, TAM, Tfh, TILs, Treg, type I IFN response, and Type II IFN response (Spearman correlation test, R > 0.20, *P* < 0.01) (Figure 6B). The *FOXH1* level showed a negative correlation with the CD8 + regulatory T cells score, cytolytic activity, NK cells, and T cell activation (Spearman correlation test, R > 0.20, *P* < 0.01). However, the *FOXD4* level was not related to the tumor immunity in the COAD (Spearman correlation test, R > 0.20, *P* < 0.01) (Figure 6B). Third, based on the level of *FOXH1 *and* FOXS1* genes, we found that the ratios of pro-/anti-inflammatory cytokines were lower in the HEG patients compared to the LEG patients (Wilcoxon sum rank test, *P* < 0.005) (Figure 6C). Fourth, we discovered a positive correlation between different immune-inhibitory hallmark markers and the level of *FOXS1* expression in COAD (Pearson’s correlation test, R > 0.20, *P* < 0.05) (Table 2). Some of the immune-inhibitory prominent marks, including *IL10, PD-1, PD-L1, PD-L2, CTLA4, HAVCR2, TIGIT, CXCL13, FAP, VIM, *and* POSTN,* were positively correlated with the expression level of *FOXS1 *(Table 2). Altogether, our immunological investigations demonstrated that the overexpressed level of *FOXH1 *and *FOXS1* is crucially related to the immunosuppression of the colon TME, which may provide a significant explanation for the oncogenic role of FOX genes.

**Table 2:** The Independent Prognostic FOX Genes Correlated with Immunosuppressive Markers in the COAD

**Immune Signature**	**Marker Gene**	***FOXD4***		***FOXH1***		***FOXS1***	
		**R**	***P***	**R**	***P***	**R**	***P***
TAM	*CCL2*	-0.06	3.10E-01	-0.14	1.85E-02	0.61	1.19E-30
	*CD68*	-0.03	6.41E-01	-0.08	1.71E-01	0.35	1.12E-09
	*IL10*	0.01	8.53E-01	-0.11	7.09E-02	0.36	5.94E-10
M2 Macrophage	*CD163*	0.04	4.67E-01	-0.14	2.06E-02	0.46	2.07E-16
	*VSIG4*	0.03	6.19E-01	-0.14	2.02E-02	0.54	6.73E-23
	*MS4A4A*	0.06	3.39E-01	-0.14	1.43E-02	0.47	7.85E-17
Treg	*FOXP3*	0.04	5.43E-01	-0.05	4.13E-01	0.38	1.72E-11
	*CCR8*	-0.02	7.17E-01	-0.07	2.54E-01	0.34	4.94E-09
	*TGFB1*	0.15	1.37E-02	-0.11	6.07E-02	0.56	8.13E-25
T cell exhaustion	*PD-1*	0.17	3.60E-03	-0.14	2.03E-02	0.21	0.000393
	*PD-L1*	0.13	2.48E-02	-0.17	3.91E-03	0.21	0.00028
	*PD-L2*	0.06	3.35E-01	-0.17	3.98E-03	0.46	3.16E-16
	*CTLA4*	0.18	2.58E-03	-0.07	2.44E-01	0.25	2.11E-05
	*HAVCR2*	0.07	2.10E-01	-0.14	1.83E-02	0.49	3.96E-19
	*TIGIT*	0.13	2.58E-02	-0.11	5.54E-02	0.24	2.76E-05
	*CXCL13*	0.13	2.42E-02	-0.19	1.19E-03	0.21	2.89E-04
	*LAYN*	-0.04	5.04E-01	-0.15	1.22E-02	0.67	2.27E-38
Monocyte	*CD86*	0.05	3.98E-01	-0.14	1.79E-02	0.45	1.73E-15
	*CD115*	0.04	5.05E-01	-0.12	4.40E-02	0.51	1.50E-20
CAFs	*COL1A1 *	0.00	9.67E-01	-0.13	2.30E-02	0.67	1.87E-39
	*COL1A2 *	-0.03	6.03E-01	-0.13	2.42E-02	0.68	2.17E-40
	*COL6A1 *	-0.02	7.49E-01	-0.17	3.04E-03	0.64	4.13E-34
	*COL6A2 *	-0.02	7.97E-01	-0.15	8.91E-03	0.70	6.93E-43
	*COL6A3 *	-0.05	4.43E-01	-0.13	2.57E-02	0.61	9.18E-31
	*CSPG4 *	-0.02	7.11E-01	-0.09	1.10E-01	0.53	2.77E-22
	*DCN *	-0.09	1.29E-01	-0.14	1.96E-02	0.64	1.58E-34
	*DES *	-0.10	9.91E-02	-0.06	3.15E-01	0.35	1.01E-09
	*FAP *	0.01	8.38E-01	-0.16	5.90E-03	0.67	1.55E-38
	*TNC *	0.00	9.81E-01	-0.18	2.63E-03	0.45	1.70E-15
	*ACTA2 *	-0.07	2.08E-01	-0.07	2.53E-01	0.73	3.17E-48
	*S100A4 *	0.00	9.83E-01	0.03	6.44E-01	0.34	2.60E-09
	*THY1 *	-0.03	5.97E-01	-0.11	5.21E-02	0.76	8.00E-55
	*VIM*	0.01	9.05E-01	-0.14	1.53E-02	0.71	8.63E-45
	*POSTN*	-0.01	8.49E-01	-0.18	2.58E-03	0.65	2.83E-35
MDSCs	*IL18BP *	0.09	1.20E-01	-0.07	2.21E-01	0.46	3.71E-16
	*FCGR2A *	0.11	7.04E-02	-0.19	1.40E-03	0.48	4.41E-18
	*FCGR2B *	0.05	3.65E-01	-0.09	1.16E-01	0.54	2.17E-23
	*FCGR3A *	0.06	2.78E-01	-0.16	7.95E-03	0.54	1.86E-23
	*ITGAL *	0.13	2.68E-02	-0.06	3.16E-01	0.33	1.39E-08
	*ITGAM *	0.09	1.42E-01	-0.11	6.70E-02	0.53	1.19E-22
	*PSAP *	0.02	7.26E-01	-0.12	3.91E-02	0.44	7.62E-15
	*S100A8 *	0.03	5.98E-01	-0.13	2.31E-02	0.33	8.48E-09
	*GPSM3 *	0.14	1.77E-02	-0.08	1.76E-01	0.41	4.14E-13
	*PARVG *	0.12	4.99E-02	-0.01	8.38E-01	0.43	1.45E-14
	*CCR2 *	0.02	7.60E-01	-0.10	9.62E-02	0.43	4.65E-14
	*CXCR4 *	0.26	1.07E-05	-0.13	2.35E-02	0.33	1.68E-08
	*FERMT3 *	0.17	4.51E-03	-0.05	3.88E-01	0.35	1.41E-09
	*CD14 *	0.12	4.79E-02	-0.15	8.78E-03	0.52	6.81E-21

R is Pearson’s correlation, and *P* is significance level.

###  Prognostic FOX Genes Associated with Cancer-associated Pathways and Biological Processes in COAD

**Figure 7:**
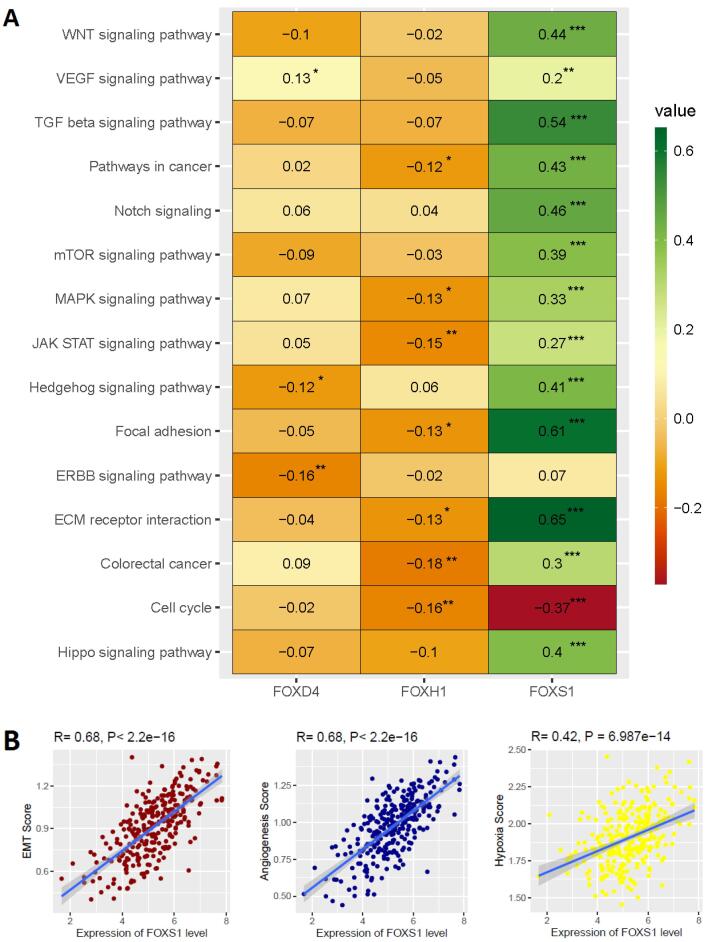
Association of Biological Signaling with the Expression of Prognostic FOX Genes in COAD**. A. **The cancer-associated pathways are correlated with prognostic FOX genes. **B.** The hallmark biological processes, including EMT, angiogenesis, and hypoxia, correlate with the expression level of *FOXS1*. * is *P* < 0.05, ** is *P* < 0.01, and *** is *P* < 0.001

 After we identified that the expression of three FOX genes (*FOXD4, FOXH1, *and* FOXS1*) are independent prognostic factors and regulate tumor immunity in COAD, we investigated the Spearman’s correlation of these three genes with various cancer-associated pathways activity (R > 0.20 and *P* < 0.01). Interestingly, we discovered a favorable correlation between the level of *FOXS1 *expression and the activities of several pathways, such as colorectal cancer, ECM receptor interaction, ERBB, Focal adhesion, Hippo, JAK-STAT, MAPK, mTOR, Notch, Pathways in cancer, TGF beta, VEGF, WNT, and Hedgehog signaling pathway (Figure 7A). In contrast, the level of *FOXS1* in COAD is inversely linked with cell cycle activity (Figure 7A). Moreover, we studied the association between the expression of these three distinct prognostic variables and biological processes associated with cancer, such as EMT, angiogenesis, and hypoxia. We revealed a favorable correlation between EMT, angiogenesis, hypoxia, and the level of *FOXS1* in COAD (Figure 7B). This implies that *FOXS1* expression crucially controls the biological processes and mechanisms involved with malignancy in COAD.

###  Genetic Alteration of Prognostic FOX Genes in Colorectal Cancer

**Figure 8:**
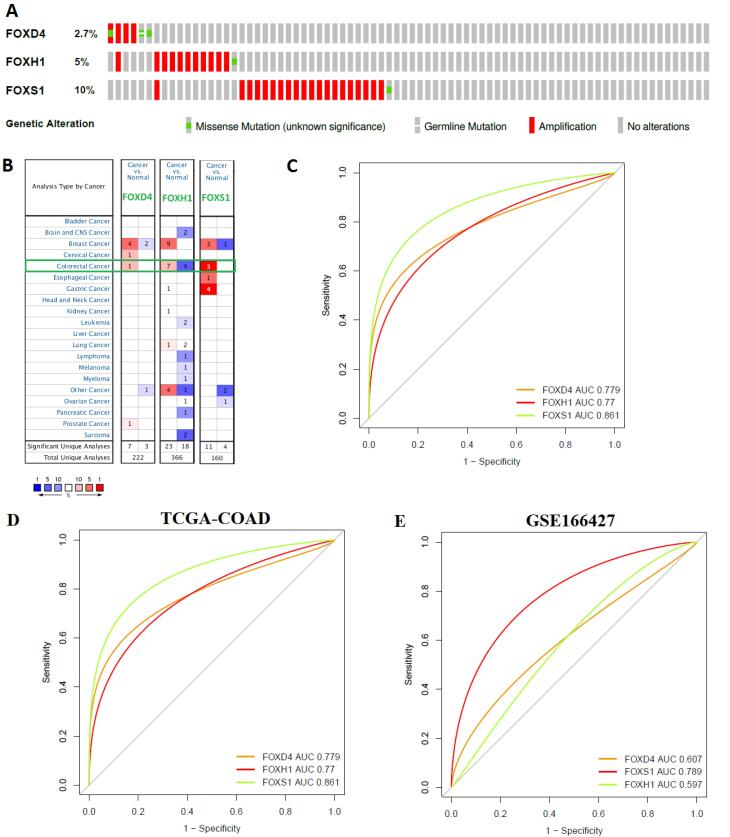
Genetic Alterations, Expression Validation, and Diagnostic Efficacy of Prognostic FOX Genes in Colorectal Cancer. **A. **Genetical alterations of *FOXD4, FOXH1, *and *FOXS1 *in TCGA COAD cohort. The *FOXD4 *(2.7%)*, FOXH1 *(5%), and *FOXS1* (10 %) genes are mutated in COAD. **B.** mRNA expression of the *FOXD4, FOXH1, *and* FOXS1* genes in different malignancies (results retrieved from the Oncomine repository). The fold change was set to 1.5, and P-value was set at 0.05. The value in the table indicates how many datasets meet the criteria. The extent of overexpression or decreased expression is linked with the strength of the color (red or blue). A light-green box indicated the mRNA deregulation of three prognostic genes. **C. **Consistent expression of *FOXD4, FOXH1, *and* FOXS1* in GSE166427**. D. **Diagnostic efficacy of three prognostic FOX genes in TCGA-COAD.We sketched the receiver operating characteristic (ROC) curve of three prognostic genes in COAD and healthy samples. The value of *FOXD4, FOXH1, and FOXS1 *revealed diagnostic efficacy in the COAD. **E.** Consistent diagnostic efficacy of three prognostic FOX genes in GSE166427^15^

 To identify the genetic changes of three prognostic FOX genes (*FOXD4, FOXH1, *and *FOXS1*), we utilized the COAD (TCGA, Firehose Legacy) datasets with mutation and CNA information (n = 220) in cBioPortal (http://www.cbioportal.org/). The 37 (17%) patient populations with the studied *FOXD4, FOXH1, *and* FOXS1* genes showed genetic alterations. We revealed that *FOXD4 *was genetically changed in 2.7% of samples,and *FOXH1 *was genetically changed in 5% of samples (Figure 8A). In addition, *FOXS1* was altered in 10% of patients (Figure 8A). The genetic changes mainly included amplification and missense mutation of three prognostic FOX genes (Figure 8A).

###  Expression Evaluation and Diagnostic Efficacy of Prognostic FOX Genes in Colorectal Cancer 

 We examined the mRNA expression of three prognostic FOX genes (*FOXD4, FOXH1, *and *FOXS1*) in a variety of malignancies, including colorectal cancer, using the Oncomine repository (https://www.oncomine.org/resource/login.html) (Figure 1 and Table 1). The value of *FOXD4, FOXH1, *and *FOXS1* were significantly up-regulated in colorectal cancer relative to the control (FC > 1.5 and *P* < 0.05) (Figure 8B). To validate the expression levels of these three genes, we used GSE166427^15^ to differentiate the expression level of *FOXD4, FOXH1, *and* FOXS1 *between the COAD and normal adjacent colon cells. Interestingly, we found that *FOXD4, FOXH1, *and* FOXS1* are consistently deregulated in GSE166427^15^ (Figure 8C).

 Since these three prognostic FOX gene members (*FOXD4, FOXH1, *and *FOXS1*) are key regulatory genes in COAD, we hypothesized that the three genes (*FOXD4, FOXH1, *and *FOXS1*) are useful for diagnosing COAD patients. We tested our stated hypothesis using the TCGA COAD dataset. For TCGA-COAD and healthy samples, the ROC curve of the expression levels of *FOXD4* (AUC = 0.779), *FOXH1* (AUC = 0.77), and *FOXS1* (AUC = 0.861) displayed remarkable diagnostic significance (Figure 8D). Also, the ROC curve using the GSE166427 showed consistent diagnostic efficacy (Figure 8E).

###  Molecular Docking Studies Identified Potential FOX Genes Interacting with Drug Compounds 

**Figure 9:**
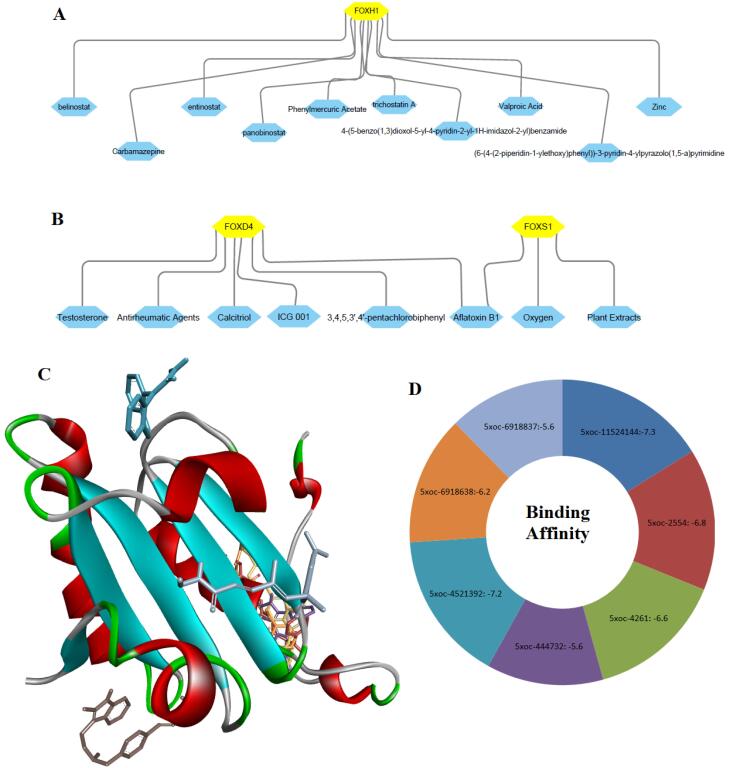
Interaction of Key Genes and Chemical Compounds.** A**. *FOXH1* interacted with various compounds. **B**. *FOXD4* and *FOXS1* interacted with some chemical compounds. **C.** Interaction of *FOXH1* (chain B of the crystal structure of human *SMAD3-FOXH1* complex: 5XOC) with chemical compounds. **D.** Binding affinity of interactions between the 5XOC (B chain of 5XOC) and chemical compounds. 5XOC: Crystal structure of human *SMAD3-FOXH1* complex; 4521392: 4- (5-benzo(1,3)dioxol-5-yl-4-pyridin-2-yl-1H-imidazol-2-yl) benzamide; 11524144: (6- (4-(2-piperidin-1-ylethoxy)phenyl))-3-pyridin-4-ylpyrazolo(1,5-a) pyrimidine; 6918638: Belinostat; 2554: Carbamazepine; 4261: Entinostat; 6918837: Panobinostat; 444732: Trichostatin A

 We inputted *FOXD4, FOXH1, *and *FOXS1 *genes into the NetworkAnalyst^32^ software for constructing the gene-compound interactions. We found that *FOXH1* interacted with 4- (5-benzo(1,3)dioxol-5-yl-4-pyridin-2-yl-1H-imidazol-2-yl) benzamide, (6- (4-(2-piperidin-1-ylethoxy)phenyl))-3-pyridin-4-ylpyrazolo(1,5-a) pyrimidine, belinostat, carbamazepine, entinostat, panobinostat, phenylmercuric acetate, trichostatin A, valproic acid and zinc (Figure 9A). Also, *FOXD4* and *FOXS1* interacted with some other compounds or agents, including 3,4,5,3’,4’-pentachlorobiphenyl, aflatoxin B1, antirheumatic agents, calcitriol, ICG 001 (Foscenvivint), oxygen (Figure 9B). Then, we downloaded the protein structure of *FOXH1* (5XOC) to identify the molecular interaction with the chemical compounds. Interestingly, we found that the *FOXH1* (chain B of the crystal structure of human *SMAD3-FOXH1* complex: 5XOC) binds with 4- (5-benzo(1,3)dioxol-5-yl-4-pyridin-2-yl-1H-imidazol-2-yl) benzamide, (6- (4-(2-piperidin-1-ylethoxy)phenyl))-3-pyridin-4-ylpyrazolo(1,5-a) pyrimidine, belinostat, carbamazepine, entinostat, panobinostat, and trichostatin A (Figure 9C). The binding affinity of these interactions is illustrated in Figure 9D.

**Figure 10:**
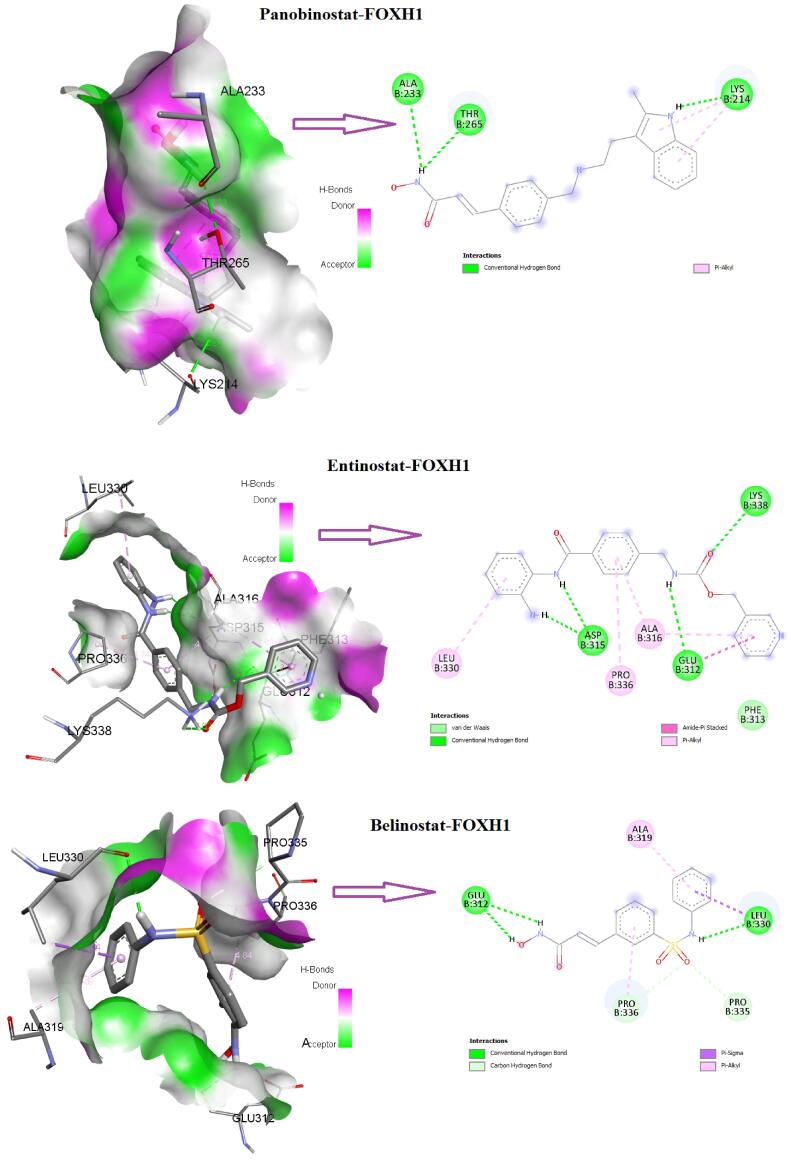
Interaction of Panobinostat, Entinostat, and Belinostat with *FOXH1* (B Chain of 5XOC) in Three-Dimensional and Two-Dimensional Approaches

Figure 10 displays the 3D and 2D interactions of *FOXH1* with the compounds that targeted it. We revealed that panobinostat interacted with *FOXH1* (The B chain of 5XOC) with an appreciable binding affinity of -5.6 kcal/mol. The three amino acid residues (LYS 214, THR 265, and ALA 233) of 5XOC interact with panobinostat (Figure 10). Moreover, the drug compound belinostat and entinostat potentially interacted with *FOXH1* (the B chain of 5XOC) (Figure 10). Also, (6- (4-(2-piperidin-1-ylethoxy) phenyl))-3-pyridin-4-ylpyrazolo(1,5-a) pyrimidine interacted with six amino acid residues (GLU 312, ASP 315, ALA 316, ALA 319, LEU 330, and PRO 336) of 5XOC. Moreover, 4- (5-benzo(1,3)dioxol-5-yl-4-pyridin-2-yl-1H-imidazol-2-yl) benzamide interacted with the ASP 315, ALA 316, ALA 319, LEU 330, and PRO 336 residues of 5XOC. Trichostatin A interacted with the TYR 281 and VAL 297, and carbamazepine interacted with ILE 271, ALA 278, ARG 284, and ILE 286. We showed that the B chain of 5XOC interacted with various drug compounds through the carbon-hydrogen bond, conventional carbon-hydrogen bond, pi-anion bond, pi-sigma bond, alkyl bond, and pi-alkyl bond. It has been stated that panobinostat is potentially used to treat colorectal cancer.^33^ Belinostat, another interacting drug, potentially altered the proteomic signatures of colon cancer cells.^34^ Entinostat combined with immunological agents reduces tumor cells in multiple murine carcinoma models.^35^ Altogether, our results suggested that these potential drug-gene interactions could be used against the malignancies of COAD.

## Discussion

 Colorectal cancer is the third leading diagnosed cancer with 9.4% of deaths worldwide.^14^ We conducted a thorough bioinformatics investigation to assess the oncogenic implications of altered FOX genes in COAD since they are linked to cancer onset, growth, advancement, migration, drug resistance, tumor immunity, and cancer-associated signaling pathways.^2-5^ Here, we explored the aberrant levels of FOX genes in COAD, the involvement of deregulated FOX genes in the functional enrichment, and the association of FOX genes in the PPI. Moreover, the FOX genes are correlated with poor clinical outcomes, tumor immunity, cancer-associated signaling pathways, and cancer hallmark biological processes. The COAD’s 32 FOX genes were deregulated (Table 1 and Figure 1). Based on earlier research, colorectal tumors frequently display different unregulated FOX genes. For example, *FOXQ1*, the top up-regulated gene with Log2FC 5.86, is up-regulated in colorectal cancer tissue samples and promotes cancer metastasis by regulating the PI3K/AKT signaling pathways.^36^ The elevated expression level of *FOXD1* was found in human CRC tissues, and *FOXD1* expression levels were correlated with tumor size and other clinical factors.^37^ *FOXA2*, another significantly upregulated gene, is overexpressed in colon cancer tissues, promotes EMT, inhibits apoptosis, and enhances the invasion ability of colon cancer cells.^38^ *FOXP2*, the top down-regulated FOX genes in COAD, promotes the invasion of hepatocellular carcinoma.^39^ Through controlling the EGFR/RAS/RAF/MEK/ERK axis, the second-ranked down-regulated gene, *FOXD3*, serves as a tumor suppressor to reduce the malignancy in colon tissue.^40^ The expression of *FOXF2* in Hela cells and tumor tissues was lower than nearby tissues, and *FOXF2* has been linked to preventing Hela cells from proliferating, migrating, and invading through controlling the Wnt biochemical signaling process.^41^ Consistent with these studies, our findings revealed that the deregulated FOX genes are crucially associated with colon carcinogenesis.

 Since the PPI network is considered an anticancer therapeutic discovery,^42^ we built the PPI network of deregulated FOX genes. Interestingly, we found the five sub-networks (Figure 3) associated with FOX genes. Sub-network 1 (Figure 3A) is crucially linked to the various cancer-associated signaling processes (Figure 3A). Numerous pathways consistently regulate colorectal cancer and other malignancies. Farhan *et al.* reported that the FOXO signaling pathways are substantial therapeutic targets in cancer treatment strategy.^43^ Uddin *et al.* established that the various pathways in cancer, small cell lung cancer, p53, apoptosis, and notch signaling processes are enriched in the colon tumor microenvironment.^44^ *FOXO* subfamily members regulate the PI3K-AKT molecular pathway.^7^ The disruption of TGF-β signaling is crucially correlated with CRC tumorigenesis and progression.^45^ Altogether, the FOX gene-associated PPI network-mediated signaling cascades may be associated with colorectal carcinogenesis.

 We identified that shorter COAD patient survival time is related to eight FOX genes (Figure 4). In numerous malignancies, the patient’s survival duration consistently correlates with the prognostic FOX genes we uncovered. For example, worse OS and DFS in colorectal cancer are linked to *FOXC1* overexpression.^46^ Individuals with colorectal cancer have a poor prognosis when *FOXD1* is up-regulated.^37^ Chen et al found that *FOXD4* activity was increased in CRC and correlated with a short survival period.^47^ Reduced lung cancer survival rates are linked to up-regulated *FOXH1* levels.^48^ *FOXQ1*, another prognostic gene, is poorly associated with prognosis in NSCLC.^49^ In patients with gastric cancer, overexpression of *FOXS1* is related to a shorter survival period.^50^ Moreover, univariate, multivariable, and nomogram analyses showed that *FOXD4, FOXH1, *and* FOXS1 *are independent prognostic markers in COAD (Figure 5). These results indicate that those with colorectal cancer have a significantly reduced survival probability due to the dysfunctional activity of FOX genes.

 The survival time of cancer patients is connected with immunogenicity, and immunological responses have a considerable effect on the clinical outcomes of patients.^51^ We evaluated the correlation of three independent prognostic FOX genes (*FOXD4, FOXH1, *and* FOXS1*) with tumor immunity in COAD (Figure 6). We found that the *FOXH1 *and* FOXS1 *genessubstantially regulate tumor immunity in COAD (Figure 6). First, the immune and stromal content are deregulated between the HEG and LEG of these FOX genes (Figure 6A). Second, these two prognostic FOX genes are significantly correlated with immune infiltrations (Figure 6B). Third, the *FOXH1* and *FOXS1* highly expressed group in COAD had considerably reduced ratios of pro-/anti-inflammatory cytokines (Figure 6C). Fourth, the *FOXH1* and *FOXS1* genes are correlated with immunosuppressive markers (Table 2). Tumor-associated macrophages (TAMs), leading immunosuppressive immune cells, stimulate CRC growth by modifying the extracellular matrix remodeling, tumor metabolism, angiogenesis, and the tumor microenvironment.^52^ In metastasis of colorectal carcinoma, M2 macrophages are associated with carcinogenesis and tumor development by enhancing the invasiveness of tumor cells.^53^ CAFs, another immunosuppressive member,^54^ improve the enrichment for TAMs and suppress NK activity in colorectal cancer.^55^ The immunosuppressive MDSCs are crucially related to the initiation and progression of CRC.^56^ The regulatory T cells (Tregs) contribute to the progression of human colorectal cancer.^57^ T cell exhaustion biomarkers, such as *TIM-3/ HAVCR2, PD-1, CTLA4,* and *TIGIT, *were overexpressed in CRC tumor tissues.^58^ Altogether, the prognostic FOX genes may be associated with the immunosuppressive colon tumor microenvironment by regulating the immune-inhibitory markers, stromal components, and immune cells.

 We also evaluated the relationships between prognostic FOX genes and pathways linked to cancer and biological processes (Figure 7). Previous studies consistently found that the correlated cancer-associated pathways are enriched in colorectal cancer. For example, Uddin *et al.* found that cancer-related signaling processes, such as ECM-receptor interaction, focal adhesion, and Wnt signaling pathway, are significantly enriched in the colon tumor microenvironment.^44^ Hedgehog signaling is associated with CRC tissue formation, proliferation, and metastasis.^59^ In addition, the hallmark biological processes of cancer, including EMT, angiogenesis, and hypoxia, regulate the initiation, development, progression, and drug resistance of colorectal malignancies.^60-62^ These suggest that the deregulated prognostic FOX genes are associated with COAD carcinogenesis by regulating the cancer-associated pathways and hallmark biological processes.

 Ultimately, we evaluated the genetic alteration and diagnostic efficacy of three independent prognostic factors (*FOXD4, FOXH1, *and* FOXS1*) in COAD (Figure 8). In addition, we investigated the expression variation of these prognostic markers in cancers using the Oncomine database and GSE166427 (Figure 8), and found consistent results which ultimately indicate the substantial roles of *FOXD4, FOXH1, *and* FOXS1 *incolon cancer. Furthermore, molecular docking studies revealed that *FOXH1* potentially interacted with various drugs (Figure 9 and Figure 10). Li et al reported that *FOXD4 *was consistently found to have diagnostic and prognostic value in colonic cancer and to be linked to the nuclear matrix, Rap1 signaling pathway, RNA transportation, and VEGF signaling framework.^63^ The growth of gastric cancer is connected with aberrantly expressed *FOXS1*, which has the potential to serve as a biomarker for both diagnosis and prognosis.^50^ Overall, these findings indicate that these FOX genes may be crucial targets for the therapeutic implications of COAD.

 Our study also has some drawbacks. First, our findings were obtained from a bioinformatics study and need experimental validation. Second, we analyzed COAD’s mRNA expression profiles, which are not equivalent to the protein expression levels. Hence, additional clinical and experimental verification will be required to convert these discoveries into practical uses for therapeutic targeting.

 In conclusion, the dysfunctional FOX genes are substantially involved with poor clinical outcomes, immunogenicity, cancer-associated pathways, and oncogenic biological processes in COAD. This study may provide novel clues for targeting the FOX genes as potential therapeutics in COAD.

## Supplementary Files

Supplementary file 1 contains the following tables:
Table S1. List of the marker genes for identifying the ssGSEA scores immune signatures, cancer-associated pathways, and hallmark biological processes.
Table S2. List of the biological processes significantly correlated to the suppressed FOX gene in COAD.
Table S3. The list of nodes in the sub-network 1.
Table S4. The list of significant KEGG pathways related to the sub-network.
